# Editorial

**Published:** 1856-06

**Authors:** 


### EDITORIAL DEPARTMENT.

Retrospect

Well, our medical missionary labors having been brought to a close for the season, we find ourselves in our *sanctum*, alone, to indulge in reminiscences of the past events. We have attended the sittings of that great *omnium gatherum* of the choice spirits of the profession, from all parts of the Union, from Maine to California, to which was added some choice material from among the subjects occupying a provincial *addendum* to the Queen's dominions. Then we have met our own brethren in our own State, at home, and here, too, were those who manifest an ardent attachment for their profession, and are emulous of rank and position in medical science. It was most gratifying to observe with what zeal each one engaged in the work of elevating the standard of the profession. We also attended an interesting meeting in a sister State,-the proceedings of which will be found in our pages.

But we find ourselves in the midst of a mass of material, which has for some time been collecting and aggregating around us. Here are books inviting our examination, a candid consideration, and then our endorsement or disapproval, as we may determine. If they are meant as bribes, we will scorn the offering, and can assure the donor and all future contributors that our opinions upon their merits will be untrammelled and unbiased, the fears of some to the contrary notwithstanding. Here, too, are scores of journals, weekly, monthly, quarterly and semi-annually, appearing, some with new, but the majority with old, familiar faces, all inviting an examination into their pages, which has been quite too long deferred. And then, here are essays, and treatises innumerable upon as many, and as great a variety of topics. Thus, at this time, we are surrounded, barricaded, beseiged, and flanked, by an accumulated and accumulating mass of heterogeneous medical literature, domestic and foreign, light and profound, brilliant and prosing, convincing and converting, old and new, good and bad, long and short, pointed and pointless, but it must be "put through" that we may be made familiar with the medical world around us, whilst we have been, truant like, away on a round of pleasure and rusticating ourselves after a long period of what might be aptly termed *hybernation* in yonder old College Halls, teaching the "young idea," the "lights and shades" of medical science, or, to speak more classically, the "*ars medendi.*"

With a sigh in undertaking our labors, and a smile at the variety thereof, (wanting in the *spice* of times) we open one Journal, look at the index, find something new which must be examined, then laid aside for future comment; then another is looked over bibliography and all, but nothing new here, saw all before; another, and another, and yet we are only beginning our labors, and so it goes on, slowly winding and wading our way to the end of the chapter.

Just in the midst of the perusal of an article which has drawn us away from the "world, time and things" and has lost us in a temporary abstraction, some messenger from the sick room, with an appeal from some poor human sufferer, dashes in upon us with a mandate imperious, and just as authoritively made known, when, overleaping the literary fortifications and embankments which we have drawn around us, we are soon away on our errand of duty.

This interruption has broken the chain of our reflections and disturbed our arrangements, and we again begin *de novo.* Among the first articles which meet the eye, is an address, in which the pursuits of the medical men are presented with that gravity, solemnity, and impressiveness, which becomes the subject, and when referring to the taste which the mind imbibes for study and the numerous sources and objects for contemplation, he quotes from the poet the following lines :

> "Tongues in trees ;
> "Books in the running brook ;
> "Sermons in stones and good in everything.

But who is the author of this excellent address? for as the first paragraph arrested our attention we were drawn unconsciously along, and neglected to note to whom belonged the credit of so able a production, and found it to be Prof. Cox before the N. Y. Medical Society.

And here is the once familiar visitant the "Scalpel" which for some time has discontinued its visits.

We have already glanced over the pages of this number, although some time has elapsed since then. We shall re-peruse it as it is unlike "Pinder's razors" in one particular—in that it was made to "cut," and not simply "to sell." Its dissections are well performed, being neat and minute, and we are pleased in being a witness to the operation. We wot of some material worthy of its metal, if not too far decomposed and softened for its nice edge. Its morbid anatomy would prove very interesting, to the moral pathologists at least. The physiology is strange and unique, judging from the manifestations daily and yearly made. A microscope would be almost needless, at most it need not multiply more than "three times" in order to a satisfactory examination.

But here is an article which has escaped our notice, and has rather a singular mast head. "Position of the Faculty and the Scalpel—Conservative Antiquaries—Fossilized Moralists—Professional Turners and their Turning Lathes—Editorial Eccentricity—Diplomas and Theories—Cribs—Tea Kettles, and Corn Baskets." We greedily perused the article, a hearty laugh followed, and we were cured of the slight ruffle which our feelings had acquired when compelled to re-commence a laborious work. The allusion to the College turning shops for the manufacture of medical *teetotums*, is too plain to be misconceived or misconstrued, and accords with our own observation, even here in the west, in a region which has received the dignified sobriquet of the "Des Moines Republic." Here an amorphous product indistinctly appeared without a kindred likeness to any-in the air, upon the earth, or in the waters, and certainly not like unto a turning "lathe" and yet manifestly the results indicated that the functions of such a piece ot contrivance were of similar attributes. It turned *over* what had been turned *out* and, as if exerting a universal and all prevalent influence and power, it *turned* out what had never been within the revolving *ma-chine* and *vice versa.*

But *turn* or change is the word, its motto, for some of its parts have always *turned*, with powers and proclivities of the fabled Isis, they are always turning or changing. To "blow hot and cold" at each revolution is a minor feat, for practice makes perfect, and for this reason this feat has become rather an automatic performance. To-day State institutions are just the thing, the desiderata; to-morrow they are fit for nothing except to make food for denunciation. One member of a ship's crew finds the craft a fine sailor, albeit, he has been all the while deranging its sails and interfering with its progress; but so soon as he is set on shore, because of his mal-management, he *turns* against the fine old ship and, because he failed to scuttle her, he changes tack and tries to get her condemned, and failing in this too, he changes again his tactics and surreptitiously tries to get on board again, but the plank was in, and he was left to fume, froth, and curse as he paced the shore, while the crew on board, despising his meanness as they defied his efforts, sneered at his folly and depravity. But true to its organization and structure it continued to *turn*—and with considerable facility. An infidel face is suddenly changed to a Janus' hypocritical expression of "sanctimonious seeming," and what could not be effected by their low means or measures may now be done under the auspices of a church—and with false assurances of belief in the faith, in piteous lamentations the cry went forth "save me or I perish," and soon we hear of the faith as profound as the bishop. Previously Catholicism had been appealed to and a partiality for its doctrines avowed.

This was a *turn* which promised to *turn* to some account. But in the very birth-time of this new-made zeal a political party sprung up whose basis lay deep down in its opposition to the before mentioned sect. Here in its midnight conclaves its influence was asked in consideration of an alliance with its organization and aid in its efforts. But we cannot now follow this *thing* through all its serpentine windings or discourse more at length of the *modus* of the working of this patent expired, univalve, illegitimate product, for it has been fully exposed by one eminently competent to analyse even such a rare mongrel specimen. The medical world through this medium has had a peep into the wonderful capacities of the *thing* for *turning* and *tumbling* both "ground and lofty" and other theatrical phantasies and puppet exhibitions, by virtue of some previous training and habits.

We have in our possession certain facts as startling as those rehearsed in "Waverly" which we shall furnish as a theme for our friend Dixen of the "Scalpel" which is so apt in the extirpation of "bodies of death" which derange and pervert the healthy vital functions. In this case the operation will cost no pain, as there is a morbid insensibility, and will require no anasthetic.

